# Profiling of N^6^-Methyladenosine (m^6^A) Modification Landscape in Response to Drought Stress in Apple (*Malus prunifolia* (Willd.) Borkh)

**DOI:** 10.3390/plants11010103

**Published:** 2021-12-30

**Authors:** Xiushan Mao, Nan Hou, Zhenzhong Liu, Jieqiang He

**Affiliations:** 1 Shandong Transport Vocational College, 7369 Bohai Road, Weifang 261206, China; maoxiushan@163.com; 2 State Key Laboratory of Crop Stress Biology for Arid Areas, Yangling, Xianyang 712100, China; nanhou@nwafu.edu.cn; 3 Shaanxi Key Laboratory of Apple, College of Horticulture, Northwest A&F University, Yangling, Xianyang 712100, China

**Keywords:** epitranscriptome, gene expression, m^6^A methylation, drought stress, apple

## Abstract

Drought stress is a significant environmental factor limiting crop growth worldwide. *Malus prunifolia* is an important apple species endemic to China and is used for apple cultivars and rootstocks with great drought tolerance. N^6^-methyladenosine (m^6^A) is a common epigenetic modification on messenger RNAs (mRNAs) in eukaryotes which is critical for various biological processes. However, there are no reports on m^6^A methylation in apple response to drought stress. Here, we assessed the m^6^A landscape of *M. prunifolia* seedlings in response to drought and analyzed the association between m^6^A modification and transcript expression. In total, we found 19,783 and 19,609 significant m^6^A peaks in the control and drought treatment groups, respectively, and discovered a UGUAH (H: A/U/C) motif. In *M. prunifolia*, under both control and drought conditions, peaks were highly enriched in the 3′ untranslated region (UTR) and coding sequence (CDS). Among 4204 significant differential m^6^A peaks in drought-treated *M. prunifolia* compared to control-treated *M. prunifolia*, 4158 genes with m^6^A modification were identified. Interestingly, a large number of hypermethylated peaks (4069) were stimulated by drought treatment compared to hypomethylation. Among the hypermethylated peak-related genes, 972 and 1238 differentially expressed genes (DEGs) were up- and down-regulated in response to drought, respectively. Gene ontology (GO) analyses of differential m^6^A-modified genes revealed that GO slims related to RNA processing, epigenetic regulation, and stress tolerance were significantly enriched. The m^6^A modification landscape depicted in this study sheds light on the epigenetic regulation of *M. prunifolia* in response to drought stress and indicates new directions for the breeding of drought-tolerant apple trees.

## 1. Introduction

According to the central dogma, RNAs are the essential and fundamental components responsible for the transfer of genetic information from DNA to proteins. During this process, over 100 distinctly chemical modifications have been reported to modify various kinds of RNAs in all living species [1]. N^6^-methyladenosine (m^6^A) RNA methylation is a crucial internal modification and is found to occur in rRNA, mRNA, tRNA, miRNA, and long non-coding RNA [2,3,4,5]. Moreover, m^6^A modification accounts for 80% of all RNA methylation modifications [6]. In 1974, m^6^A was discovered as a dominant type of mRNA methylation in mammals for the first time [7]. Nowadays, m^6^A modifications have been widely reported in various species, such as viruses, plants, yeast, humans, and other mammals [3,5]. Among plants, m^6^A was identified in wheat (*Triticum turgidum* L.), oat (*Avena sativa* L.), and maize (*Zeamays* L.) about 40 years ago [8,9,10]. m^6^A is a dynamic and reversible modification process that requires three effectors: “writer”, “reader”, and “eraser” proteins. Writers carry out m^6^A modification, readers recognize the methylation, and erasers demethylate m^6^A modifications [11,12]. In the current study, writer protein complexes include METTL3, METTL14, WTAP, etc.; readers are mainly the YTH-domain-containing proteins; and erasers include FTO and ALKBH5 [13,14,15,16]. Additionally, studies have shown that m^6^A is involved in nuclear–cytoplasmic export, RNA stability, pre-mRNA splicing, primary microRNA processing, alternative polyadenylation site choice, and translation efficiency in mRNA metabolic processes [17,18,19,20,21,22].

Recently, with the development of m^6^A sequencing (m^6^A-seq) technology, an increasing number of comparative m^6^A methylome studies have been conducted to better understand its role in plant biological processes. In *Arabidopsis thaliana*, m^6^A regulates leaf morphology [23], trichome development [24], floral transition [25], and embryonic development [22]. In addition, m^6^A regulates microspore degeneration in rice [26] and is responsible for the fruit ripening of tomato [27] and strawberry [28]. Stress responses are also affected by m^6^A modification. In *Arabidopsis*, YTH-domain proteins evolutionarily conserved the C-terminal region 1 (ECT1) and ECT2 which interact with calcineurin B-like-interacting protein kinase1 (CIPK1) and mediate calcium signaling under various stresses [29]. Compared with wild-type *Arabidopsis*, the *alkbh6* mutant plants exhibit low survival rates under abiotic stresses, including salt, drought, and heat stresses [30]. Increased levels of m^6^A methylation have been observed in rice plants’ response to viral infection [31]. In maize, m^6^A hypomethylation under drought stress has a favorable function in drought response [32]. The function of m^6^A modification in pak choi (*Brassica rapa ssp. chinensis*) under heat stress has also been investigated [33]. A recent study on apple found that the m^6^A reader YTH domain-containing RNA binding protein 2 (YTP2) regulates *Mildew Locus O 19* (*MdMLO19*) mRNA stability and translation efficiency of antioxidant genes to confer powdery mildew resistance [34]. Although m^6^A has been reported in both biotic and abiotic stresses, its role in drought stress in non-model plants is currently unknown.

Extreme climate change causes frequent global droughts and high temperatures, which severely limit crop growth and yield [35]. Apple is one of the world’s popular fruits; however, its production and quality are frequently threatened by drought stress [36,37]. Apple propagation mainly relies on vegetative propagation via grafting and budding. *M. prunifolia* is a wild relative of apple with strong biotic and abiotic resistance to drought, cold, heat, and disease [38], which makes it one of the best rootstocks in northwest China, where apple cultivars are usually grafted to vigorous rootstocks, including *M. prunifolia* and *Malus sieversii.* In addition, *M. prunifolia* is commonly used as a parent in cross breeding studies for stress tolerance [39]. Despite the outstanding performance of *M. prunifolia* in improving apple drought tolerance, the molecular mechanism of *M. prunifolia* in response to drought is largely unclear.

In this study, we first performed a transcriptome-wide m^6^A modification profile of *M. prunifolia* seedlings and investigated changes in m^6^A modification after drought stress. We also performed an RNA-seq analysis and identified differentially expressed genes (DEGs) in *M. prunifolia* in response to drought. To investigate the potential relationship between m^6^A levels and gene expression levels in *M. prunifolia* in response to drought stress, we performed association analysis between differential m^6^A peaks and DEGs. Our data allowed the identification of some drought-responsive genes along with changes in gene expression by m^6^A modifications in *M. prunifolia* after drought stress, such as *Heat shock protein 60* (*HSP60*), *jasmonate-Zim-domain protein 3* (*JAZ3*), *Scarecrow-Like 1* (*SCL1*), and *ETHYLENE RESPONSE FACTOR1 (ERF1).* Overall, our work illustrates the m^6^A modification landscape of *M. prunifolia* in response to drought stress and provides new insights into the molecular mechanisms operating in conditions of drought.

## 2. Materials and Methods

### 2.1. Plant Materials and Stress Treatment

One-year-old *M. prunifolia* seedlings were used as the plant materials. Seeds of *M. prunifolia* ‘Fupingqiuzi’ were collected from Fuping (Weinan, Shannxi, China) and stratified in wet sand at 4 °C for three months. Then, the seeds were sowed in a plant growth chamber with 8000 lux light intensity, 14 h light/10 h dark photoperiods at 25 °C. Three months later, the *M. prunifolia* seedlings were moved to a greenhouse at the Northwest Agriculture and Forest University, Yangling (34°20′ N, 108°24′ E), Shaanxi Province, China. The seedlings were transplanted into plastic pots (15 cm × 20 cm, ~1.3 L) filled with a mixture of garden soil and substrate (PINDSTRUP, Denmark) (1:1, *v*/*v*). The stress treatment was started a year later, when the seedlings were 1.5 m tall. Forty-two seedlings with uniform growth were chosen and every seventh seedling were used as a biological replicate. When the treatment began, all the seedlings were watered until saturated (control) and then water was withheld from half the plants until the relative soil water content reached approximately 40% (drought treatment). Mature leaves were collected from the middle of the trees for the following RNA extraction. 

### 2.2. RNA-Seq Analysis

Leaves were collected from control and drought-treated *M. prunifolia* seedlings. Total RNA was extracted using the cetyltrimethylammonium bromide (CTAB) method according to a previously described procedure [40]. The RNA-seq library was constructed as previously reported by Xie et al. [41]. RNAs were subjected to sequencing on the Illumina HiSeq 4000 platform by Novogene (Beijing, China). Sequences were aligned to a recently released *Malus × domestica* genome sequence (GDDH13 version 1.1, https://iris.angers.inra.fr/gddh13/downloads/GDDH13_1-1_formatted.fasta.bz2, accessed on 18 November 2021.) [42] using HISAT2 v2.1.0. BAM conversion, sorting, and indexing were performed using SAMtools v1.9. Read counting within genes wase analyzed with HTSeq v0.12.4 using the gene annotation file (https://iris.angers.inra.fr/gddh13/downloads/gene_models_20170612.gff3.bz2, accessed on 18 November 2021.) [43]. Differences in gene expression were analyzed by DESeq2 v1.30.1 with a threshold of an adjusted *p*-value below 0.05 and |log2(fold change)| greater than 1 [44]. Length of genes were calculated by GenomicFeatures v1.42.3, and fragments per kilobase of transcript per million fragments mapped (FPKM) values were obtained by TBtools [45,46]. Heatmaps of gene expression levels were plotted using pheatmap v1.0.12 [47]. Gene Ontology (GO) enrichment analyses were performed using agriGO v2.0 and clusterProfiler v.3.18.1 [48,49]. 

### 2.3. m^6^A-Seq Analysis

mRNA m^6^A was sequenced by MeRIP-seq at Novogene (Beijing, China). Briefly, a total of 300 μg RNA was extracted from the leaves. The integrity and concentration of extracted RNAs were detected using an Agilent 2100 bioanalyzer (Agilent, Santa Clara, CA, USA) and simpliNano spectrophotometer (GE Healthcare, Chicago, IL, USA), respectively. Fragmented mRNA (~100 nt) was incubated for 2 h at 4 °C with anti-m^6^A polyclonal antibody (Synaptic Systems, Göttingen, Germany) in the immunoprecipitation experiment. Then, immunoprecipitated mRNAs or Input was used for library construction with NEBNext ultra-RNA library prepare kit for Illumina (New England Biolabs, Ipswich, MA, USA). The library preparations were sequenced on an Illumina Novaseq platform with a paired-end read length of 150 bp according to the standard protocols. The sequencing was carried out with three independent biological replicates.

Raw reads from m^6^A-seq were trimmed to remove adaptor sequences and bases with a quality lower than 20 using Trimmomatic v0.39 and FastQC v0.11.9 [50,51]. The remaining reads were mapped onto the apple reference genome by HISAT2 v2.1.0 [52]. Post-processing was carried out by SAMtools v1.9 [53]. Peak calling was analyzed by exomePeak2 v1.2.0 with a *p*-value below 0.05 and a log2(fold change) greater than 1 [54]. The other running parameters of exomePeak2 were set as: fragment_length = 100, binding_length = 25, step_length = 25, peak_width = 50. The overlapping peaks of each biological replicate and a Venn diagram were generated by intervene v0.6.5 [55]. FindMotifsGenome.pl in HOMER v4.10.0 was employed to identify the m^6^A motifs [56]. Differentially methylated peaks were identified using exomePeak2 with a threshold of an adjusted *p*-value below 0.05 and |DiffModLog2FC| above 0.5. The CMRAnnotation tool in PEA v1.1 and bedtools were used to annotate the peaks’ different transcript distributions using the gene annotation file [57,58]. The visualization of m^6^A peaks was performed using the Integrative Genomics Viewer v2.10.2 [59].

## 3. Results

### 3.1. Transcriptome-Wide Mapping of m^6^A in Malus prunifolia Seedlings

**Figure 1:**
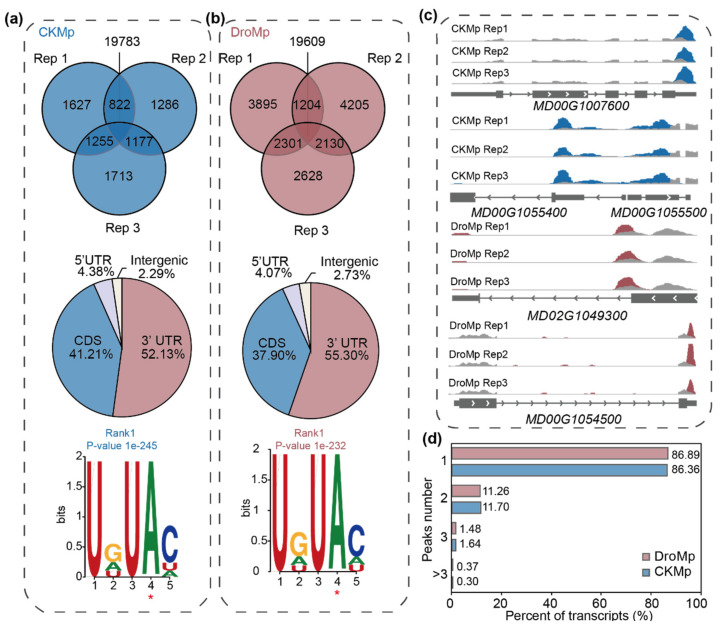
Transcriptome-wide m^6^A methylome in *Malus prunifolia* seedlings in response to drought. (**a**,**b**) Venn diagrams demonstrating the overlap of m^6^A peaks from three replicates (**top**), the enriched motif (**bottom**), and m^6^A peak distribution along transcripts (**middle**) under control (**a**) or drought conditions (**b**). The asterisks in the two motifs marked the positions that are modified. CDS, coding sequence; UTR, untranslated region; DroMp, *M. prunifolia* seedlings were treated with drought stress; CKMp, *M. prunifolia* seedlings were grown under control conditions. (**c**) Integrative Genomics Viewer (IGV) tracks. (**d**) Percentage of m^6^A-containing transcripts with m^6^A peaks in DroMp and CKMp.

To investigate whether m^6^A methylation participates in drought stress in apples, we constructed and sequenced a series of m^6^A-immunoprecipitation (IP) and matched input libraries to obtain the drought and control-treated *M. prunifolia* transcriptome-wide m^6^A maps. Each library was prepared with three biological replicates. Pearson correlation coefficient analysis among biological replicates showed reliable repeatability (Figure S1 in Supplementary Materials). As shown in Table S1, we generated a total of 23–28 million reads for each m^6^A-seq sample and 21–32 million reads for each input sample (Table S1). The proportion of clean and mapped reads in m^6^A-seq were around 52–77%. Transcriptome-wide m^6^A modification sites were identified using exomePeak2. After m^6^A peak calling analysis, we identified 19,783 and 19,609 common peaks in *M. prunifolia* under control and drought stress conditions, respectively (Table S2 and Table S3 in Supplementary Materials). The proportions of common peaks in *M. prunifolia* under control conditions were above 80% (84.23%, 85.64%, and 83.04%) (Figure 1a), while the proportions of common peaks in *M. prunifolia* under drought conditions were all around 73% (72.97%, 72.20%, and 73.81%) (Figure 1b). In order to estimate the accuracy of peaks, we randomly selected five m^6^A-containing genes from peaks of *M. prunifolia* under control and drought stress conditions by checking the read abundance in the Integrative Genomics Viewer (IGV) (Figure 1c). In *M. prunifolia* under control conditions, m^6^A modifications on *MD00G1007600*, *MD00G1055400*, and *MD00G1055500* were modified in the 3′ untranslated region (UTR), the 5′ UTR, and the coding sequence (CDS), respectively. In *M. prunifolia* under drought conditions, m^6^A modifications on *MD02G1049300* and *MD00G1054500* were modified in the CDS and 3′ UTR (Figure 1d). These results indicated that the m^6^A-seq data are reliable.

To acquire a better understanding of the m^6^A distribution pattern in *M. prunifolia* under control conditions and drought stress, we evaluated the distribution of m^6^A in the whole transcriptome. The transcripts were divided into three non-overlapping regions: 5′ UTR, CDS, and 3′ UTR. As shown in Figure 1a,b, m^6^A modifications in *M. prunifolia* under control and drought conditions were mainly enriched in 3′ UTR, followed by CDS, with a small amount of enrichment in the 5′ UTR and intergenic regions (Figure 1a,b). The m^6^A distribution pattern in *M. prunifolia* under drought stress has a 3.17% greater proportion in the 3′ UTR than that under control conditions, as well as a 3.31% lower percentage in the CDS. Nevertheless, m^6^A maintained the same distribution trend in control- and drought-treated *M. prunifolia*, that is, m^6^A peaks were mainly distributed in the 3′ UTR, followed by the CDS.

Previous work has demonstrated that multiple different regions of a transcript may undergo m^6^A modification [28]; we therefore calculated the number of peaks in each transcript. As shown in Figure 1d, about 86% m^6^A-modified transcripts contained one m^6^A peak, about 11% contained two m^6^A peaks, and only a few contained more than three m^6^A peaks. The trends were almost identical in *M. prunifolia* under control and drought conditions, similar to those in strawberry and pak choi [28,33].

Furthermore, we used the HOMER software to investigate the m^6^A modification motif in *M. prunifolia* under control and drought conditions. Results showed that the UGUAH (H: A/U/C) sequence motif is the predominant and conserved sequence in *M. prunifolia* under control and drought conditions (Figure 1a,b). In the HOMER results, the motif containing UGUAH is ranked first in *M. prunifolia* under control and drought conditions with a *p*-value of 1e-245 and 1e-232, respectively (Figure 1a,b). The UGUAH motif was consistent with findings in *Arabidopsis* [24], tomato [28], and maize [32]. However, the conserved m^6^A modification motif RRACH (R: A/G; H: A/U/C) was also discovered in *Arabidopsis* [25,60,61], indicating that the pattern of m^6^A modification varies among species.

**Figure 2:**
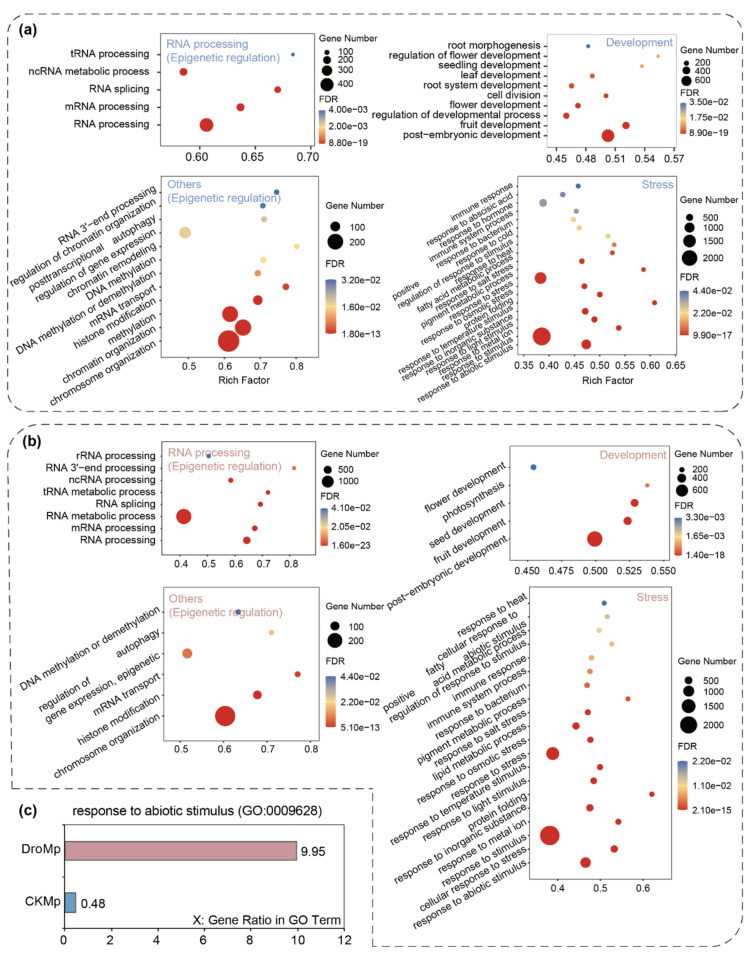
Gene ontology (GO) enrichment analysis of m^6^A-modified genes in *Malus prunifolia* seedlings in response to drought. (**a**,**b**) The “biological process” aspect of the GO enrichment analysis was divided into “RNA processing (epigenetic regulation)”, “others (epigenetic regulation)”, “development”, and “stress”. (**c**) The gene ratio of “response to abiotic stimulus (GO:0009628)” in *Malus prunifolia* seedlings in response to drought conditions. DroMp, *M. prunifolia* seedlings were treated with drought stress; CKMp, *M. prunifolia* seedlings were grown under control condition; FDR, false discovery rate.

Since m^6^A methylation has been widely reported to be involved in regulating biological processes in plants [24,25,28,31,33,62], we performed gene ontology (GO) enrichment analyses of m^6^A-containing genes in control- and drought-treated *M. prunifolia* seedlings. As shown in Figure 2a,b, the m^6^A-containing genes in *M. prunifolia* under control and drought were significantly enriched in a number of pathways: (1) RNA processing: RNA processing, RNA splicing, ncRNA (metabolic) processing, and tRNA (metabolic) processing; (2) others: RNA 3′-end processing, mRNA transport, histone modification, regulation of gene expression, chromosome (chromatin) organization, and DNA methylation or demethylation; (3) development: flower development, fruit development, and post-embryonic development; (4) stress: response to abiotic stimulus, response to osmotic stress, response to heat, response to temperature stimulus, response to salt stress, response to stimulus, response to abiotic stimulus, response to metal ion, immune response, protein folding, and fatty acid metabolic process. Notably, the m^6^A-containing genes under drought conditions showed a higher proportion in response to abiotic stimulus (GO: 0009628) than those under control conditions in *M. prunifolia* (Figure 2c). These results indicated not only that m^6^A is widely involved in various biological processes in *M. prunifolia* but implied that m^6^A modification in *M. prunifolia* is responsive to abiotic stimulus after drought treatment.

### 3.2. Differential m^6^A Methylation between Control and Drought-Treated M. prunifolia Seedlings

**Figure 3:**
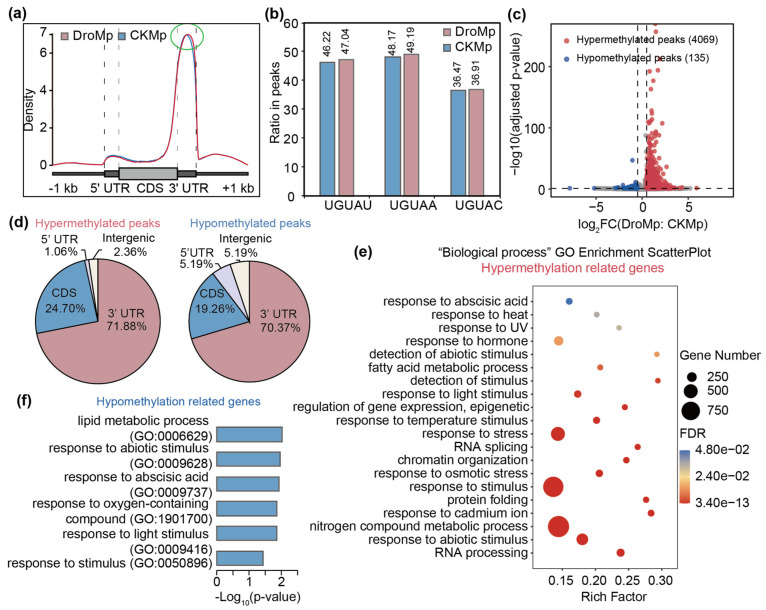
Differential m^6^A modifications in *Malus prunifolia* seedlings in response to drought. (**a**) Density of m^6^A peak distribution along transcripts of *Malus prunifolia* seedlings in response to drought conditions. The area circled in green shows the difference in density between the two groups. (**b**) Percentage of UGUAH (H: A/U/C). (**c**) Volcano plot showing hypermethylated and hypomethylated m^6^A peaks. (**d**) Pie charts showing the m^6^A peaks distribution within transcripts. (**e**) GO enrichment analysis of hypermethylated peak-related genes. (**f**) GO enrichment analysis of hypomethylated peak-related genes. DroMp, *M. prunifolia* seedlings were treated with drought stress; CKMp, *M. prunifolia* seedlings were grown under control condition; CDS, coding sequence; UTR, untranslated region; FDR, false discovery rate.

To observe the changes of m^6^A methylation in *M. prunifolia* seedlings after drought treatment, we produced m^6^A distribution plots (Figure 3a) using GuitarPlot [63] and a histogram of UGUAH statistics in peaks (Figure 3b. The transcript features in m^6^A distribution plots included upstream 1 kb, 5′ UTR, CDS, 3′ UTR, and downstream 1 kb. As shown in Figure 3a, the densities of m^6^A peaks were mainly enriched in the 3′ UTR in *M. prunifolia* under control and drought treatment, though we noticed a slightly increased peak density in *M. prunifolia* under drought conditions (Figure 3a). After calculating the percentage of the subsequences of the UGUAH motif (UGUAU, UGUAA, and UGUAC) under control and drought conditions, we found that UGUAA and UGUAU were mainly enriched in *M. prunifolia*. Compared to control-treated *M. prunifolia*, the proportion of all three subsequences of UGUAH in drought-treated *M. prunifolia* showed a slight increase. Our results suggested that the modification patterns of m^6^A did not change in *M. prunifolia* after the drought treatment.

To gain better insight into the potential roles of m^6^A in regulating drought resistance in *M. prunifolia*, we next focused on the differential m^6^A peaks with thresholds of |log2 (fold change) | > 0.5 and adjusted the *p*-value < 0.05 by comparing the m^6^A methylome of *M. prunifolia* under drought and control conditions (Table S4 in Supplementary Materials). After drought treatment, 4069 peaks were up-regulated and 135 peaks were down-regulated, corresponding to 4026 and 135 transcripts, showing that more peaks were hypermethylated under drought stress in *M. prunifolia* (Figure 3c). We then assigned the differential m^6^A peaks to transcript features (Figure 3d). As shown in Figure 3d, the 4069 hypermethylated m^6^A peaks were highly enriched in the 3′ UTR (71.88%) and CDS (24.70%); similarly, the 135 hypomethylated peaks were also mainly distributed around the 3′ UTR (70.37%) and CDS (19.26%). These results are consistent with the increased m^6^A peak density in 3′ UTR in *M. prunifolia* under drought conditions (Figure 3a). To further explore the biofunctional aspects of these hypermethylated and hypomethylated genes, GO enrichment analysis was performed. For the hypermethylated genes, most genes were significantly enriched in stress and stimulus-related GO slims, such as response to abiotic stimulus, abscisic acid (ABA), heat, temperature stimulus, osmotic stress, and protein folding. Other GO slims that exhibited an association with epigenetic regulation included RNA processing and splicing, chromatin organization, and gene expression regulation. Additionally, hypomethylated genes were mainly enriched in the lipid metabolic process (GO:0006629), response to abiotic stimulus (GO:0009628), response to abscisic acid (GO:0009737), response to oxygen-containing compounds (GO:1901700), response to light stimulus (GO:0009416), and response to stimulus (GO:0050896). These data imply that the m^6^A levels of some genes in response to drought, including those responsive to ABA, may be influenced by the drought treatment.

### 3.3. Differential Gene Expression Analysis

**Figure 4:**
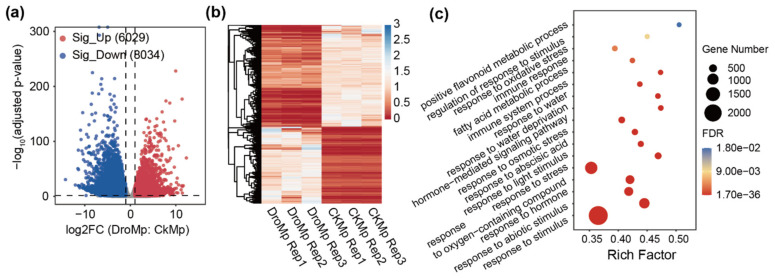
Differential gene expression in *M. prunifolia* in response to drought treatment. (**a**) Volcano plot showing up-regulated genes and down-regulated genes in *M. prunifolia* after drought treatment. (**b**) Heat map of differentially expressed genes (DEGs). (**c**) GO enrichment analysis of DEGs. Sig_Up, up-regulated genes; Sig_Down, down-regulated genes; DroMp, *M. prunifolia* seedlings were treated with drought stress; CKMp, *M. prunifolia* seedlings were grown under control condition; FDR, false discovery rate.

*M. prunifolia* is known for its tolerance of harsh environments and is particularity adapted to drought stress [38]. To profile the gene expression changes regulated by drought stress in *M. prunifolia*, we performed differential gene expression analysis using RNA-seq data. The differentially expressed genes (DEGs) were identified with thresholds of |log2 (fold change)|> 1 and an adjusted *p*-value < 0.05 by comparing the reads of *M. prunifolia* under control and drought conditions using the DESeq2 package (Table S5 in Supplementary Materials) [44]. As shown in Figure 4a, the volcano plot showed that 6029 genes were up-regulated in *M. prunifolia* under drought stress compared with the control condition, while 8034 genes were down-regulated. The heatmap also displayed the same results using fragments per kilobase of exon model per million mapped reads (FPKM). GO enrichment analysis revealed that DEGs significantly concentrated in relation to three aspects of GO slims: (1) metabolic processes: the positive flavonoid metabolic process and fatty acid metabolic process; (2) hormones: the hormone-mediated signaling pathway, response to abscisic acid, and response to hormone; and (3) stress: (positive regulation of) response to stimulus, response to abiotic stimulus, response to osmotic stress, response to water (deprivation), response to oxidative stress, and immune response. These data showed that *M. prunifolia* underwent dramatic and significant changes in the expression levels of a large number of drought-related genes after drought stress, which may be related to the drought resistance of *M. prunifolia*.

### 3.4. Association Analysis of m^6^A Levels with Gene Expressions Involved in Apple Drought Tolerance

**Figure 5:**
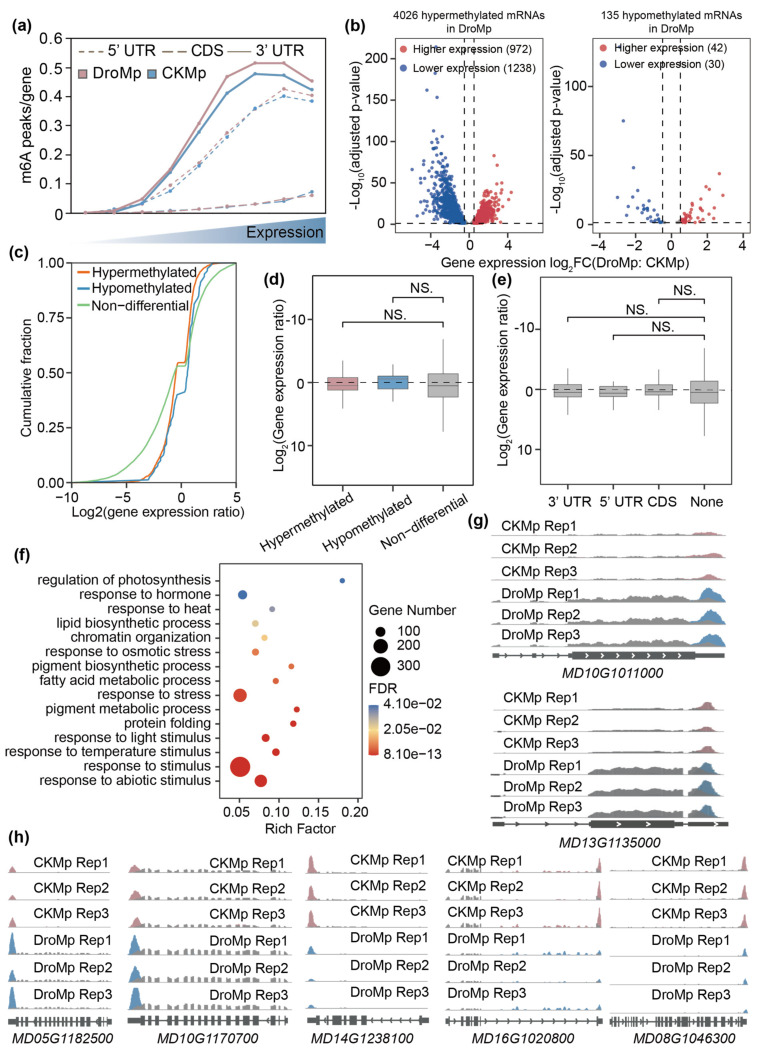
Correlation between m^6^A modification levels and mRNA abundance in *M. prunifolia* in response to drought treatment. (**a**) The ratio of m^6^A peaks in different transcript distributions to total transcripts in each subgroup was divided by the FPKM. (**b**) Volcano plots displaying the gene expression ratios of hypermethylated and hypomethylated transcripts. (**c**) Cumulative fraction of mRNA expression changes. (**d**) Box plot of gene expression ratios in hypermethylated, hypomethylated, and non-differential transcripts. Hypermethylated, all differentially expressed genes (DEGs) with hypermethylation; hypomethylated, DEGs with hypomethylation; non-differential, DEGs without m^6^A modification. (**e**) Box plot of gene expression ratios in CDS, 3′ UTR, 5′ UTR, and non-differential transcripts. None, DEGs without m^6^A modification. (**f**) GO enrichment analysis of the overlapping genes between DEGs and differentially modified m^6^A peak-related genes in *M. prunifolia* after drought treatment. (**g**,**h**) IGV tracks showing the m^6^A read distribution in drought-related genes from (**b**) and (**c**). DroMp, *M. prunifolia* seedlings were treated with drought stress; CKMp, *M. prunifolia* seedlings were grown under control condition; CDS, coding sequence; UTR, untranslated region.

As a common regulatory mechanism, m^6^A modification regulates gene expression in a wide range of biological processes [64]. In order to estimate the relationship between the m^6^A modification and gene expression levels, we divided the genes into nine groups according to FPKM from low to high as well as into three categories based on transcript distribution, and calculated the fraction of m^6^A-containing genes in each group (Figure 5a). As shown in Figure 5a, the m6A peak fraction increased in the 5′ UTR, 3′ UTR, and CDS with increasing gene expression levels. The highest density was at the seventh group for 3′ UTR, the eight group for CDS, and the ninth group for 5′ UTR. Overall, the m^6^A peak fraction between control and drought treatment showed little variation in the CDS and 5′ UTR, but more m^6^A modifications were correlated with higher gene expression levels in 3′ UTR under drought conditions.

To investigate the potential relationship between m^6^A levels and gene expression levels in response to drought stress, we performed association analyses between differential m^6^A peaks and DEGs. As shown in Figure 5b, two volcano diagrams showed the overlapping of methylated genes and DEGs. In hypermethylated genes, 972 genes were up-regulated and 1238 genes were down-regulated in *M. prunifolia* under drought stress. In hypomethylated genes, 42 and 30 genes showed higher and lower expression levels in *M. prunifolia* under drought stress, respectively. We then extended this analysis to the entire transcriptome of all m^6^A-modified genes (Figure 5c,d). Genes bearing hypermethylation and hypomethylation exhibited no significant expression changes compared with the non-differential m^6^A-modified genes according to a Wilcoxon test (Figure 5d). Considering the transcript distribution characteristics in m^6^A methylation, we analyzed the gene expression changes in the whole transcriptome (Figure 5e). However, statistical analysis indicated that different transcript distributions did not significantly affect differential gene expression compared with non-differential m^6^A genes (Figure 5e). These data imply a complex relationship between m^6^A levels and expression levels.

To further understand the DEGs affected by m^6^A modifications, we performed GO enrichment analysis. The results revealed that these genes were significantly enriched in stress-related GO slims, such as lipid and pigment biosynthetic processes, the fatty acid metabolic process, protein folding, response to hormone, response to heat, response to osmotic stress, and response to temperature stimuli (Figure 5f). Chromatin organization, which plays a role in plant responses to drought, was also significantly enriched [65]. Based on this GO enrichment analysis, several DEGs affected by m^6^A modifications were exhibited in IGV [59]. Two up-regulated *Heat shock protein 60* (*HSP60*) genes (*MD05G1182500*, *MD10G1170700*) were hypermethylated in *M. prunifolia* under drought stress (Figure 5h). HSPs are essential components of thermotolerance in plants and *HSP60* is reported to be up-regulated in *Arabidopsis* after high temperature stress [65,66]. Two hypomethylated *jasmonate-Zim-domain protein 3* (*JAZ3*) genes (*MD14G1238100*, *MD16G1020800*) were down-regulated in *M. prunifolia* under drought stress, consistent with results in poplar [67].

It has been reported that the establishment of stress acclimation and stress adaptation associates with changes in genome DNA methylation and may depend on small RNA pathways requiring *Dicer-like 2* (*DCL2*) and *DCL3* [68,69]. We also found down-regulated *DCL3* with hypomethylation in *M. prunifolia* under drought stress. The *Arabidopsis ETHYLENE RESPONSE FACTOR1 (ERF1)*-overexpressing plants (*35S:ERF1*) are more tolerant to drought and salt stress compared with wild-type plants [70]. Under drought stress, *ERF1* (*MD13G1135000*) in *M. prunifolia* showed hypermethylation and up-regulated gene expression. Additionally, as shown in Figure 5g, hypermethylated *Scarecrow-Like 1* (*SCL1*) exhibited up-regulated gene expression in *M. prunifolia* after drought stress. *Ct-SCL1* has been reported to play a key role in the survival of the cluster bean (*Cyamopsis tetragonaloba* L.) under drought stress by interacting with the SWITCH SUBUNIT 3B (SWI3B) protein through chromatin remodeling and stress-based epigenetic memory [71]. The above data suggested that m^6^A modifications are widely involved in the response to drought in *M. prunifolia* by affecting the expression of drought-related genes.

## 4. Discussion

The m^6^A modification pattern has been reported in many plant species. In different ripening stages of strawberry, m^6^A peaks are mainly modified in the 3′ UTR and stop codon, followed by the CDS, and there was no significant change in the ratio of the three regions [28]. In maize, m^6^A peaks frequently occur in the 3′ UTR (around 70%), followed by about 20% in the stop codon [32,72]. This trend is also observed in *Arabidopsis* [25,73]. During tomato fruit ripening, m^6^A peaks are mainly modified in the 3′ UTR and stop codon, and the percentage of 3′ UTR modifications increases with fruit ripening [27]. In pak choi (*Brassica rapa* ssp. *chinensis*), m^6^A peaks are mainly enriched in the 3′ UTR and CDS with approximate proportions which show no change after heat stress [33]. Therefore, m^6^A frequently occurs in th 3′ UTR or stop codon. In contrast, a recent report on apple shows m^6^A peaks mainly enriching the CDS (53.20%), followed by the 3′ UTR (36.80%) and 5′ UTR (10.00%) [34]. The transcript distribution annotations of peaks are based on the apple gene annotation file without region information for start and stop codons (GDDH13 version 1.1, https://iris.angers.inra.fr/gddh13/downloads/gene_models_20170612.gff3.bz2, accessed on 18 November 2021) [42]. Here, we extracted the region information for the 3′ UTR, CDS, and 5′ UTR from the gene annotation file and completed the transcript annotation using middle point of peaks (probably the strongest point of m^6^A modifications) [32,74]. Peaks that do not overlap at all with the three regions are defined as intergenic peaks. Our results showed that m^6^A peaks were mainly enriched in the 3′ UTR in *M. prunifolia* under control and drought conditions. The difference between the two results in apple may be caused by different annotation methods. As shown in Figure 5g, m^6^A modification of *MD09G1253000* in the 3′ UTR might also occur in the stop codon region. Interestingly, we noticed that only about 60% of the transcripts had 3′ UTR and about 36% of the transcripts did not have either 5′ UTR and 3′ UTR information in the gene annotation file during the process of peak annotation. These data further indicate that incomplete information in the apple gene annotation file and different annotation methods may lead to different m^6^A distribution results in apple.

m^6^A is widely conserved among eukaryotes and tends to occur in the RRACH (R: A/G; H: A/U/C) consensus motif, which was identified in mammals in the 1970s [75]. In the early days of m^6^A research in *Arabidopsis*, RRACH was also identified [61]. However, a later study in *Arabidopsis* identified the conserved UGUAY (Y: C/U) motif [23]. In addition, in maize and tomato, the conserved m^6^A motif was identified as UGUAMM (M = A or C) and UGUAYY, respectively [27,32]. In our results, the UGUAH motif ranked first in the HOMER [56] results and was highly significant, similar to the “URUAY” motif reported by Guo et al. [34]. Moreover, our results also showed that UGUAU and UGUAA had the highest percentage (Figure 3b). The m^6^A conserved motif in *M. prunifolia* is relatively consistent with that in *Arabidopsis*, maize, and tomato. These data indicate not only that UGUA is the core sequence of m^6^A modification in plants but also show the complexity and bias of m^6^A modification among different species. 

There have been several reports on m^6^A modifications and their effects on mRNA abundance in plants in response to biotic and abiotic stresses. In pak choi, more m^6^A modifications show hypermethylation after heat treatment, but m^6^A affected the same number of up- and down-regulated genes [33]. In apple, *MhYTP2* overexpression enhanced apple powdery mildew (PM) resistance and triggered more hypermethylated peaks [34]. Similarly, our results showed that a large number of hypermethylated peaks appeared in *M. prunifolia* under drought stress. To further investigate the potential relationship between differential m^6^A peaks and DEGs, we considered the role of methylation types and transcript distribution in m^6^A modifications and performed an analysis mentioned only in a study of strawberry fruit ripening [28]. However, no significant or apparent correlation was found between the differential deposition of m^6^A in gene features and altered gene expression. We speculated that this may be related to the different organ or biological process in our study.

Abiotic stresses adversely affect plant growth and productivity. Numerous studies have been conducted to decipher the genetic and molecular mechanisms of plant drought stress tolerance [76,77]. Members of the heat shock protein (HSP) family remodel proteins and play various positive roles in plant responses to drought stress. *HSP20* was up-regulated under high-temperature stress in pepper and grasses [78,79]. In tobacco, *NtHSP70-1* was found to be an ABA-inducible gene, and the over-expressed *NtHSP70-1* can confer drought stress tolerance [80]. In *Arabidopsis thaliana*, the overexpression of *AtHsp90.2*, *AtHsp90.5*, and *AtHsp90.7* enhanced plant responses to drought and salt stresses, and cytosolic Hsp90 might be involved in plant stress responses in an ABA-dependent manner [81]. In our results for m^6^A-modified genes with expression changes in drought-treated *M. prunifolia* compared to control-treated *M. prunifolia*, we found a number of up-regulated *HSP* genes encoding HSP20, HSP70, HSP90.5, HSP88.1, HSP90.6, and HSP60. Further, it is well known that ABA mediates the drought stress response by regulating stomatal closure and stress-responsive gene expression [82,83]. SNF1-ralated protein kinases 2 (SnRK2s) are key regulators that manage the adaptive responses to osmotic stress, including drought stress [84]. SNRK2.6, a member of subclass III, plays essential roles in the positive regulation of ABA signaling and is strongly activated by ABA [85]. ERF1 integrates JA, ET, and ABA signaling through stress-specific gene regulation and plays a positive role in salt, drought and heat stresses [70]. In *Arabidopsis*, the overexpression of *ERF1* enhanced drought and salt tolerance [70]. Similar to our results, hyper-methylated *SNRK2.6* and *ERF1* were up-regulated in *M. prunifolia* after drought treatment as compared to control-treated *M. prunifolia*. These m^6^A-modified genes with altered gene expression in HSP encoding genes and the ABA pathway of *M. prunifolia* after drought treatment establish a link between m^6^A modification and drought tolerance in apple.

In summary, we depicted a transcriptome-wide m^6^A profiling in *M. prunifolia* and investigated changes in m^6^A modification after drought stress. We found that drought stimulated hypermethylated m^6^A peaks in *M. prunifolia*. Several m^6^A-modified drought-responsive genes, including *HSP60, JAZ3*, *SCL1*, and *ERF1*, were presented. Our research provides new support for understanding the epigenetic regulatory mechanisms of apples in response to drought stress and the breeding of drought-tolerant apple trees.

## Data Availability

The RNA-seq and m^6^A-seq data have been deposited with the NCBI with the dataset identifier PRJNA781274.

## Supplementary Materials

The following supporting information can be downloaded at: https://www.mdpi.com/article/10.3390/plants11010103/s1, Figure S1: The Pearson correlation analysis of m^6^A-seq and RNA-seq data, Table S1: Summary of sequenced and mapped reads in m^6^A-seq and RNA-seq samples generated in this study, Table S2: m^6^A-modified genes in *M. prunifolia* seedlings under control conditions, Table S3: m^6^A-modified genes in *M. prunifolia* seedlings under drought conditions, Table S4: Differential m^6^A peaks in drought-compared to control-treated *M. prunifolia*, Table S5: Differentially expressed genes in drought-versus control-treated *M. prunifolia*.
