# Supplementary material for: Profiling of N6-Methyladenosine (m6A) Modification Landscape in Response to Drought Stress in Apple (Malus prunifolia (Willd.) Borkh)
Source: Plants (Basel). 2021 Dec 30;11(1):103. doi: 10.3390/plants11010103 (PMC8747461; doi:10.3390/plants11010103)
Supplement: Supplementary file 1 [file plants-11-00103-s001.zip › Supplementary Figure S1.pdf]

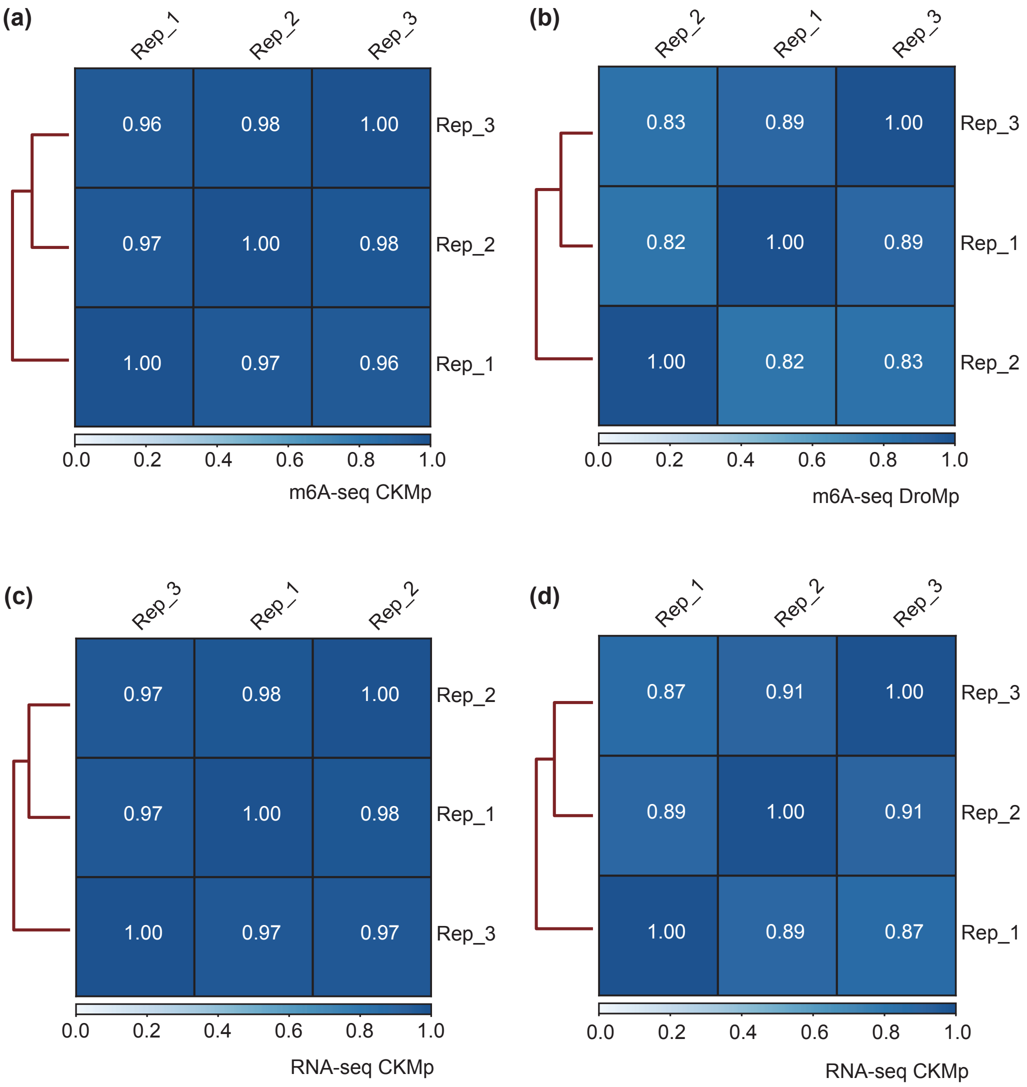

Supplementary Figure S1. The Pearson correlation analysis of m^{6}A-seq and RNA-seq data. (a) m^{6}A data of three biological replicates in *M. prunifolia* under control condition. **(b)** m^{6}A data of three biological replicates in *M.prunifolia* under drought condition. (c) RNA-seq data in *M.prunifolia* under ccontrol condition. **(d)** RNA-seq data in *M. prunifolia* under drought condition. CKMp, *M. prunifolia* seedlings under control condition; DroMp, *M. prunifolia* under drought condition.
